# Focus on shared decision‐making

**DOI:** 10.1111/hex.12654

**Published:** 2017-11-16

**Authors:** Carolyn A. Chew‐Graham

**Affiliations:** ^1^ Primary Care and Health Sciences Keele University Staffordshire UK; ^2^ South Staffordshire and Shropshire Healthcare NHS Foundation Trust Staffordshire UK

Welcome to this edition of Health Expectations, which has a focus on shared decision‐making (SDM) and the importance of health literacy in health‐care access and provision.

Health literacy is the degree to which individuals have the capacity to obtain, process and understand basic health information and services needed to make appropriate health decisions. As Rowlands and Protheroe^1^ reported in 2015, there is a mismatch between the complexity of health materials and the skills of the English adult working‐age population. Those most in need of health information have the least access to it. They suggest that efficacious strategies including building population skills through education, improving health professionals’ communication skills and written health information.

Resonating with this message, Thompson et al, in their qualitative study, interviewing older men and adopting a narrative analysis approach, report a number of influences on health literacy and vulnerability in relation to food and health for older bereaved men. The authors acknowledge the limitations of their study, recruiting as they did, men from more affluent backgrounds, all being White British and retired from professional and managerial roles, potentially implying a reasonable level of health literacy. The authors recognize the need for further research with men who may be more at risk of food vulnerability.

Chinn's paper reminds us that individually tailored information is more likely to meet personalized health information needs for people with intellectual disabilities. The emergence of different social formations in the creation of accessible information has potential for advancing engagement of diverse groups. Webster and colleagues’ survey leads them to suggest that current use of verbal descriptors to communicate side‐effect risk in patient information leaflet (PILs) leads to high side‐effect expectations. They suggest that expectations could contribute to nocebo‐induced medication side effects experienced by patients. Additional work is required to identify ways to improve the way risk information is conveyed in PILs. The need for such PILs to take account of health literacy is obvious.

Muscat and co‐authors describe how SDM programmes can be designed in a way that both support teachers to deliver novel health literacy content and empower learners. They suggest that there is benefit in fostering collaboration between adult education and health‐care sectors to reach disadvantaged populations who are rarely the focus of SDM interventions, but who stand to benefit the most from them.

According to NICE (National Institute for Health and Care Excellence in England),^2^ SDM starts with the conversation between the person receiving care and the person delivering care. SDM puts people at the centre of decisions about their own treatment and care by:

1. Exploring care or treatment options and their risks and benefits.
2. Discussing choices available.
3. Reaching a decision about care or treatment, together with their health‐ and social care professional.

Stiggelbout et al^3^ stress the need for SDM not to be seen as a “tedious added extra”, but as the core of good clinical practice, with patients placed fully at the centre of all decisions. Land and colleagues report that even for non‐negotiable treatment trajectories, the spirit of SDM can be invoked through practices that encourage participation including bringing the patient towards shared understanding of the rationale for treatment. Goodwin's study, however, reports a qualitative study which identified a mismatch between the perspectives of patients with cancer and physicians managing them: some patients felt their physicians did not assess the level of information they were able or wished to receive, and most mentioned that information provided was not helpful. In contrast, most physicians interviewed perceived that the information they provided to patients was sufficient and easy to understand.

Kinsey and colleagues provide evidence for the use of “Option Grids” to support SDM within consultations, reporting that the Option Grid for osteoarthritis of the knee was well received by patient participants, who reported that it helped them to understand their options, and made the notion of choice explicit.

Brabers et al^4^ examined the relationship between health literacy and self‐reported patient involvement and suggested a positive significant association between the health literacy scale appraisal of health information and self‐reported involvement in key decisions. They recommend that further research is needed to unravel the relationship between health literacy and patient participation in SDM. HEX would welcome manuscripts which help to respond to this research question.
